# Overhydration, Cardiac Function and Survival in Hemodialysis Patients

**DOI:** 10.1371/journal.pone.0135691

**Published:** 2015-08-14

**Authors:** Mihai Onofriescu, Dimitrie Siriopol, Luminita Voroneanu, Simona Hogas, Ionut Nistor, Mugurel Apetrii, Laura Florea, Gabriel Veisa, Irina Mititiuc, Mehmet Kanbay, Radu Sascau, Adrian Covic

**Affiliations:** 1 Department of Nephrology, University of Medicine and Pharmacy “Gr. T. Popa”, Iasi, Romania; 2 Department of Cardiology, University of Medicine and Pharmacy “Gr. T. Popa”, Iasi, Romania; 3 Department of Medicine, Division of Nephrology, Koc University School of Medicine, Istanbul, Turkey; Scuola Superiore Sant'Anna, ITALY

## Abstract

**Background and objectives:**

Chronic subclinical volume overload occurs very frequently and may be ubiquitous in hemodialysis (HD) patients receiving the standard thrice-weekly treatment. It is directly associated with hypertension, increased arterial stiffness, left ventricular hipertrophy, heart failure, and eventually, higher mortality and morbidity. We aimed to assess for the first time if the relationship between bioimpedance assessed overhydration and survival is maintained when adjustments for echocardiographic parameters are considered.

**Design, setting, participants and measurements:**

A prospective cohort trial was conducted to investigate the impact of overhydration on all cause mortality and cardiovascular events (CVE), by using a previously reported cut-off value for overhydration and also investigating a new cut-off value derived from our analysis of this specific cohort. The body composition of 221 HD patients from a single center was assessed at baseline using bioimpedance. In 157 patients supplemental echocardiography was performed (echocardiography subgroup). Comparative survival analysis was performed using two cut-off points for relative fluid overload (RFO): 15% and 17.4% (a value determined by statistical analysis to have the best predictive value for mortality in our cohort).

**Results:**

In the entire study population, patients considered overhydrated (using both cut-offs) had a significant increased risk for all-cause mortality in both univariate (HR = 2.12, 95%CI = 1.30–3.47 for RFO>15% and HR = 2.86, 95%CI = 1.72–4.78 for RFO>17.4%, respectively) and multivariate (HR = 1.87, 95%CI = 1.12–3.13 for RFO>15% and HR = 2.72, 95%CI = 1.60–4.63 for RFO>17.4%, respectively) Cox survival analysis. In the echocardiography subgroup, only the 17.4% cut-off remained associated with the outcome after adjustment for different echocardiographic parameters in the multivariate survival analysis. The number of CVE was significantly higher in overhydrated patients in both univariate (HR = 2.46, 95%CI = 1.56–3.87 for RFO >15% and HR = 3.67, 95%CI = 2.29–5.89 for RFO >17.4%) and multivariate (HR = 2.31, 95%CI = 1.42–3.77 for RFO >15% and HR = 4.17, 95%CI = 2.48–7.02 for RFO >17.4%) Cox regression analysis.

**Conclusions:**

The study shows that the hydration status is associated with the mortality risk in a HD population, independently of cardiac morphology and function. We also describe and propose a new cut-off for RFO, in order to better define the relationship between overhydration and mortality risk. Further studies are needed to properly validate this new cut-off in other HD populations.

## Introduction

Cardiovascular events (CVE), mostly related to hypertension and left ventricular hypertrophy (LVH), are the main cause of the increased mortality observed in hemodialysis (HD) patients. Chronic subclinical volume overload occurs very frequently and may be ubiquitous in HD patients receiving the standard thrice-weekly treatment. It is directly associated with hypertension, increased arterial stiffness, LVH, heart failure, and eventually, higher mortality and morbidity [1].

Traditionally, “dry weight” was achieved in hemodialysis by using trial and error clinical methods [2]. However, such an empiric approach rarely solves the problems of hypertension, intradialytic hypotension and subclinical overhydration. Although probing for the lowest tolerated post-dialysis weight improved hypertension and survivalin the setting of low sodium, long-hours slow ultrafiltration dialysis [3, 4], such results are more difficult to obtain in every center, with daily standard clinical practice, and are accompanied by frequent hypotension and low quality of life [5]. Recently, bioimpedance devices have become available for routine practice, showing similar abilities in assessingan adequate “dry weight” as the probing methodperformed by the same experience Tassin clinicians [6].

Accumulating evidence suggests that a strict bioimpedance guided fluid management has a beneficial impact on blood pressure, arterial stiffness, LVH and survival [7–10]. However, the best cut-off point for defining overhydration has yet to be proved. Furthermore, different bioimpedance derived parameters have been used—absolute fluid overload (AFO), relative fluid overload (RFO), time averaged fluid overload (TAFO)–with different cut-off points being proposed to define overhydration (eg. 1.1 L for AFO, 15% for RFO) [11, 12].

Therefore, we conducted a prospective trial to investigate in a HD cohort the impact of overhydration on all-cause mortality and CVE, by using a previously reported cut-off value for overhydration (a RFO of 15%) and also investigating a new cut-off value derived from our analysis in this specific cohort. Most importantly, we aimed to assess for the first time if the relationship between bioimpedance assessed overhydration and these outcomes is maintained when adjustments for echocardiographic parameters are considered.

## Methods

### 1. Patients

The protocol of this study was approved by the Ethics Committee of University Hospital ‘Dr C.I.Parhon’ (Iasi, Romania) and all included patients signed an informed consent. Between May 2008 and December 2010, we invited all patients (N = 298) undergoing chronic HD treatment for at least 3 months in the “Dr. C. I. Parhon” hemodialysis unit to take part in this study. Due to technique limitations, bioimpedance was not performed in patients with metallic joint prostheses (N = 11), cardiac pacemakers (N = 8), decompensated cirrhosis (N = 5) and limb amputations (N = 13). Other exclusion criteria were refusal to take part in the study, age<18 years old, active systemic infections and terminal illnesses (N = 40). Details of the final patient population (N = 221) are presented in Table 1. HD therapy was performed 4 h × three times per week, using high-flux Fresenius Polysulfone membrane dialyzers.

**Table 1:** Demographic characteristics and bioimpedance assessment of the entire study population and of the overhydrated and normohydrated patients (using the RFO = 15% cut-off).

	All patients (N = 221)	RFO ≤ 15% (N = 162)	RFO > 15% (N = 59)	P^#^
Age, years	53.8±13.9	53.5±13.7	54.6±14.4	0.59
Male, N (%)	116 (52.5)	78 (48.1)	38 (64.4)	**0.03**
Dialysis vintage, months	83.0 (49.0–130.5)	70.8 (45.8–121.9)	107.9 (74.5–150.3)	**<0.001**
Diabetes, N (%)	23 (10.4)	14 (8.6)	9 (15.3)	0.15
BMI, kg/m^2^	25.5±5.0	25.9±5.3	24.2±3.9	**0.01**
Hypertensive, N (%)	145 (65.6)	104 (64.2)	41 (69.5)	0.46
SBP, mmHg	143.1±15.7	142.4±15.7	145.0±15.9	0.27
DBP, mmHg	80.5±10.3	80.5±10.6	80.3±9.7	0.90
CV comorbidities, N (%)	112 (50.7)	82 (50.6)	30 (50.8)	0.98
CAD, N (%)	50 (22.6)	41 (25.3)	9 (15.3)	0.11
PVD, N (%)	28 (12.7)	21 (13.0)	7 (11.9)	0.83
Heart failure, N (%)	71 (32.1)	52 (32.1)	19 (32.2)	0.99
Stroke, N (%)	13 (5.9)	8 (4.9)	5 (8.5)	0.34
AFO, L	1.7±1.4	1.0±0.9	3.4±0.8	**<0.001**
RFO, L	9.7±8.1	6.0±5.8	19.7±3.6	**<0.001**
TBW, L	33.9±6.1	33.8±6.0	34.5±6.1	0.40
ECW, L	16.4±2.9	15.9±2.9	17.5±2.9	**0.001**
ICW, L	17.6±3.4	17.8±3.4	17.1±3.5	0.18
LTI, Kg/m^2^	12.6±2.5	12.7±2.6	12.2±2.5	0.22
FTI, Kg/m^2^	11.2 (7.6–15.4)	12.3 (8.4–16.0)	10.0 (6.9–13.4)	**0.01**
Deaths, N (%)	66 (29.9)	39 (24.1)	27 (45.8)	**0.002**
CVE, N (%)	78 (35.3)	46 (28.4)	32 (54.2)	**<0.001**

Data are expressed as mean ± SD, median with IR, or total number with percentages, as appropriate. Bold values are statistically significant. AFO–absolute fluid overload; BMI–body mass index; CAD–coronary artery disease; CV–cardiovascular; CVE–cardiovascular events; DBP–diastolic blood pressure; ECW–extracellular water; FTI–fat tissue index; ICW–intracellular water; LTI–lean tissue index; PVD–peripheral vascular disease; RFO–relative fluid overload; SBP–systolic blood pressure; TBW–total body water.

^#^—comparison between groups.

### 2. Bioimpedance analysis

The **body composition** was determined before dialysis using the portable whole-body multifrequency bioimpedance analysis device (Body Composition Monitor–BCM). The technique involves attaching electrodes to the patient’s non-fistula forearm and ipsilateral ankle, with the patient in a supine position. The BCM measures the body resistance and reactance to electrical currents of 50 discrete frequencies, ranging between 5 and 1000 kHz. Based on a fluid model using these resistances, the extracellular water (ECW), the intracellular water (ICW) and the total body water (TBW) are calculated. These volumes are then used to determine the amount of fluid overload. The software of the BCM device automatically performs all calculations. AFO is defined as the difference between the expected patient’s ECW under normal physiological conditions and the actual ECW, whereas the RFO is defined as the absolute fluid overload to extracellular water ratio (AFO/ECW). Patients were considered overhydrated using a cut-off of >15% for the relative hydration status. This definition of overhydration is based on the work described by Wabel et al. [11] and Wizemann et al. [12]. Using a different statistical approach (see bellow) we determined another cut-off for defining overhydration (RFO>17.4%). From the BCM data, we also obtained lean tissue index (LTI–lean mass normalized to body surface area) and fat tissue index (FTI–fat mass normalized to body surface area).

### 3. Echocardiography

Echocardiographic evaluations were performed after a short dialysis period. All echocardiographic measurements were carried out according to the recommendations of the American Society of Echocardiography [13] by a cardiologist blinded to the bioimpedance results. Due to the refusal of the patient (N = 50) and poor echocardiographic window (N = 14) we were able to perform an echocardiography assessment in only 157 patients of the entire study population (the echocardiographic subgroup).

Predialysis **blood pressure (BP) was defined** as an average BP measurement from the previous three consecutive HD sessions. The BP was measured after ten minutes of recumbence, using a standard mercury sphygmomanometer, with cuffs of appropriate size, in the arm without arterio-venous fistula. We defined hypertension as a predialysis BP >140/90 mmHg or the use of antihypertensive medications.

### 4. Outcome

The main outcomes were all-cause mortality and CVE. Secondary outcomes considered in the study were hospitalizations for any reason and hospitalizations for decompensated heart failure. Patients were censored at the last follow-up (31 May 2014) or if they moved to another dialysis unit, switch to peritoneal dialysis or received a kidney transplant. Information on fatal and nonfatal CVE including death, stroke, and myocardial infarction were obtained from the Dialysis Unit registries.

### 5. Statistical analysis

Continuous variables were expressed as mean ± SD or median and inter-quartile range (IR) according to normal or non-normal distribution. The normality of the distribution was assessed by the Shapiro-Wilk test. Categorical variables were expressed as percentages. Between-group comparisons were performed for the nominal variables with the *Χ* ^2^ test, and by Mann-Whitney test or independent T-test for the remaining variables, as appropriate.

The association between RFO and all-cause mortality was investigated by Kaplan–Meier analysis and Cox regression analysis. To determine the optimal cut-off point for the RFO as a predictor of all-cause mortality the relationship between RFO and outcome was analyzed using the Martingale residuals in Cox’s proportional hazard regression analysis [14]. We found that the categorization of RFO into two categories (<17.4% and >17.4%) was the best approach for modeling this relationship. In the multivariate Cox models we included all the available variables that are known to influence all-cause mortality in HD patients: age, gender, dialysis vintage, diabetes, cardiovascular comorbidities, hypertension, and, in the echocardiographic subgroup, left ventricular mass index (LVMI) or left ventricular ejection fraction (LVEF). For the Cox regression analysis performed in the echocardiographic subgroup, due to the low number of incident outcomes, we determined the CIs for estimating β by bootstrapping. Proportional hazards assumptions were tested using log minus log survival plots and Schoenfeld residuals. The Bayesian information criterion (BIC) and the Akaike information criterion (AIC) were calculated for each Cox model; there is no statistical test that compares different BIC or AIC estimations, and a lower value indicates a better fitted model.

We also performed a post-hoc power analysis (Rosner B. Fundamentals of Biostatistics. 7th ed. Boston, MA: Brooks/Cole; 2011.). In the entire cohort, our study had more than 80% power to detect a significant difference in mortality for both cut-offs (85.7% for the 15% RFO cut-off and 96.2% for the 17.4% RFO cut-off, respectively) at a 2 tailed-alpha of 0.05. However, in the echocardiographic subgroup, this analysis showed a 61.7% power for the 15% RFO cut-off and a 65.7% power for the 17.4% RFO cut-off, respectively, at a 2 tailed-alpha of 0.05.

The level of significance was set to *P* = 0.05. All statistical analyses were performed using the SPSS software, version 19.0.1 (SPSS Inc., Chicago, IL, USA).

## Results

### 1. Analysis of the entire cohort

Two hundred twenty one patients were included in the final analysis: 52.5% males, mean age 53.8±13.9 years, median dialysis vintage of 83.0 (IR = 49.0–130.5) months; the most frequent cause of end-stage renal disease was chronic glomerulonephritis (38.9%). 59 (26.7%) patients were considered overhydrated when judged by the RFO = 15% cut-off. There were no significant differences in age, systolic and diastolic BP, or incidence of diabetes and cardiovascular comorbidities between patients with RFO >15% and patients with RFO ≤15%. However, there were significant differences between the two groups regarding dialysis vintage and, as expected, other bioimpedance parameters—Table 1.

**Fig 1:**
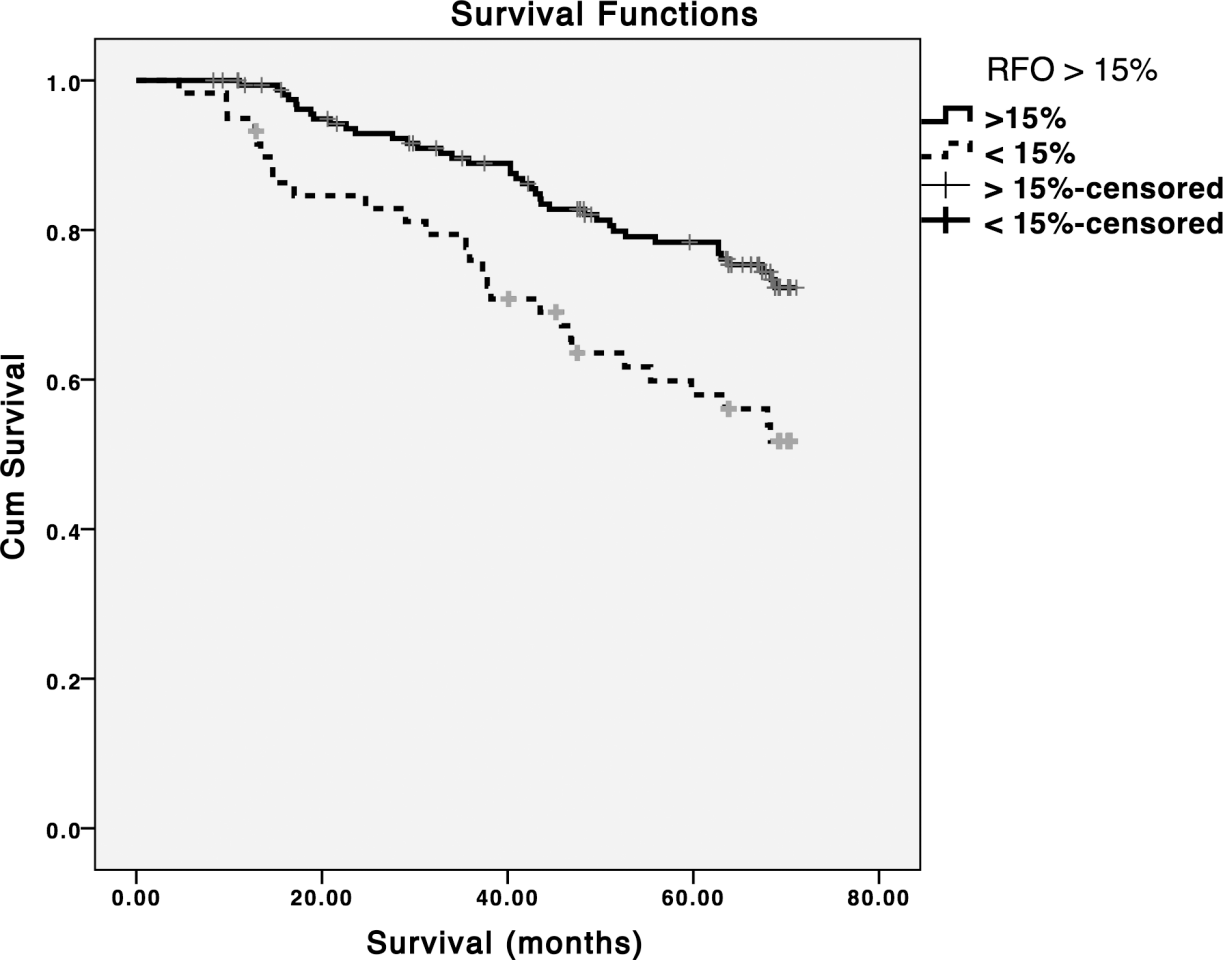
Kaplan Meier survival analysis for a RFO > 15% cut-off point (Log rank p = 0.002).

**Fig 2:**
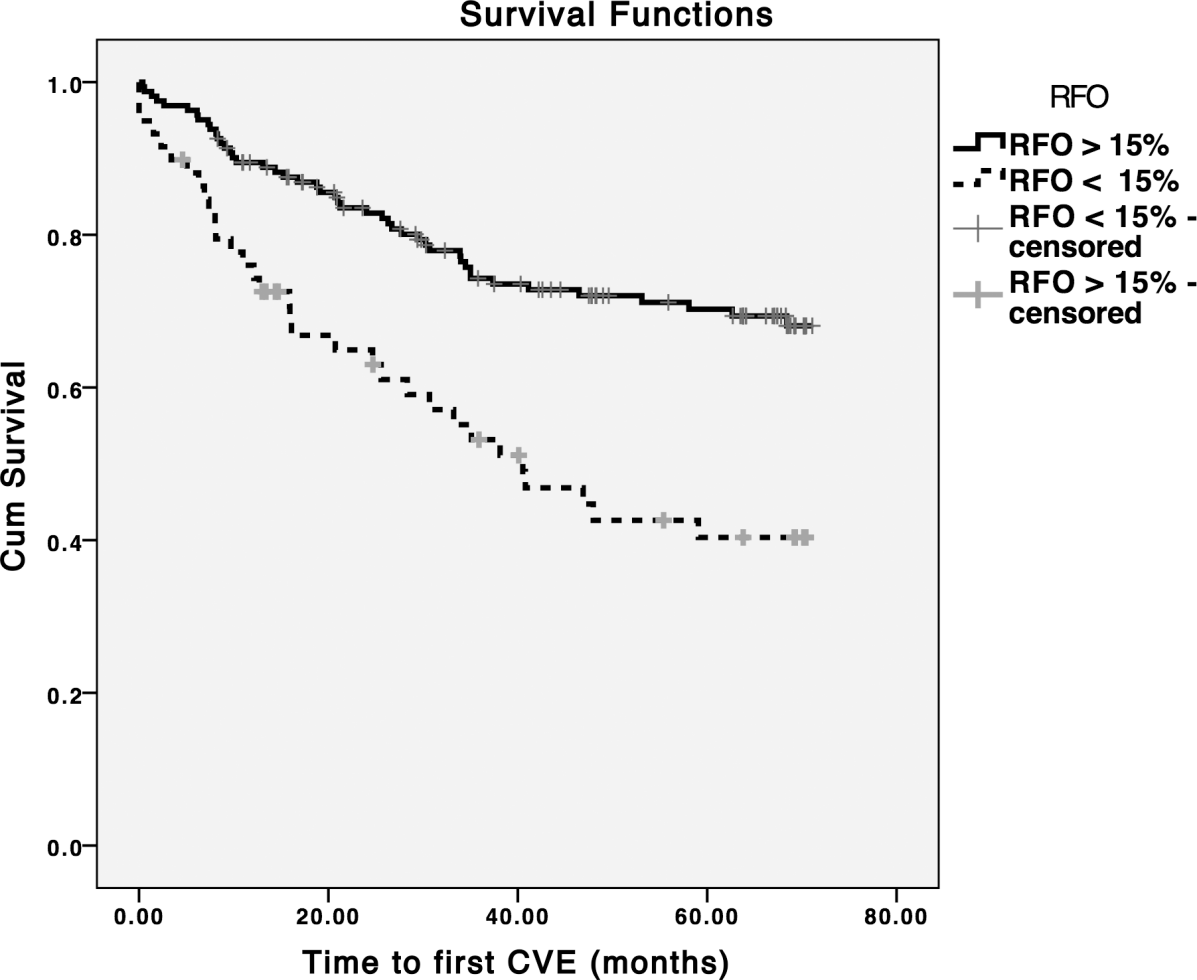
Time to first cardiovascular event analysis for a RFO > 15% cut-off point (Log rank p < 0.001).

During a median follow-up period of 66.2 (IR = 42.4–70.2) months, 66 deaths and 78 CVE were recorded. The exact causes of death are presented in the S2 Table. Patients in the overhydrated group (RFO>15%) had a 2.12 and 2.46 fold increased risk for all-cause mortality and CVE, respectively (Table 2 and Figs 1 and 2). In the multivariable Cox analysis, after adjustment for age, gender, dialysis vintage, diabetes, cardiovascular comorbidities and hypertension, a RFO>15% remained independently associated with both outcomes (Table 2).

**Table 2:** Survival analysis using the predefined cut-off of RFO (15%).

	All-cause mortality		Cardiovascular events	
	HR^a^	95% CI	HR^a^	95% CI
Unadjusted	2.12	1.30–3.47	2.46	1.56–3.87
Adjusted^b^	1.87	1.12–3.13	2.31	1.42–3.77

a. The group of patients with a RFO≤15% was used as reference.

b. Adjusted for: age, gender, dialysis vintage, diabetes, cardiovascular comorbidities, and hypertension.

The demographic, clinical characteristics and bioimpedance-derived parameters of the population categories using the Cox defined cut-off (RFO = 17.4%) are shown in the S1 Table. Patients considered overhydrated using this cut-off (RFO>17.4%) had an increased risk for both outcomes as compared to patients considered normohydrated, both in the univariate and in the multivariate survival analysis after adjustment for age, gender, dialysis vintage, diabetes, cardiovascular comorbidities, and hypertension (Table 3). However, the Cox models obtained using the RFO = 17.4% cut-off showed a better goodness of fit than the models that were obtained using the RFO = 15% cut-off (**all-cause mortality**–for the univariate analysis: AIC = 663.26, BIC = 664.82 vs. AIC = 668.78, BIC = 670.34; for the multivariate analysis: AIC = 652.06, BIC = 662.98 vs. AIC = 658.81, BIC = 669.73; **CVE**–for the univariate analysis: AIC = 762.87, BIC = 764.43 vs. AIC = 773.56, BIC = 775.12; for the multivariate analysis: AIC = 748.84, BIC = 759.77 vs. AIC = 763.87, BIC = 774.79).

**Table 3:** Survival analysis using the new cut-off of RFO (17.4%).

	All-cause mortality		Cardiovascular events	
	HR^a^	95% CI	HR^a^	95% CI
Unadjusted	2.86	1.72–4.78	3.67	2.29–5.89
Adjusted^b^	2.72	1.60–4.63	4.17	2.48–7.02

a. The group of patients with a RFO≤17.4% was used as reference.

b. Adjusted for: age, gender, dialysis vintage, diabetes, cardiovascular comorbidities, and hypertension.

Analyzing the all-cause hospital admissions, only the patients found overhydred by the new cut-off showed a significant increase in the incidence rate for this outcome (Table 4). However, the hydration status (considered by either of the two cut-offs) was not associated with an increase in the incident rate for decompensated heart failure hospitalizations (Table 4).

**Table 4:** Outcome data for the entire cohort–hospitalizations.

	RFO≤15% (N = 162) (751.2 Patient-Years at Risk)		RFO>15% (N = 59) (251.1 Patient-Years at Risk)		Rate Ratio (95%CI)	RFO≤17.4% (N = 181) (852.7 Patient-Years at Risk)		RFO>17.4% (N = 40) (155.6 Patient-Years at Risk)		Rate Ratio (95%CI)
	No. of events	No. of events/100 patient-years	No. of events	No. of events/100 patient-years		No. of events	No. of events/100 patient-years	No. of events	No. of events/100 patient-years	
All-cause hospitalizations	456	60.2	180	71.7	0.84 (0.71–1.01)	515	60.4	121	77.8	0.78 (0.64–0.95)
Decompensated heart failure hospitalizations	19	2.5	12	4.8	0.53 (0.24–1.19)	23	2.69	8	5.14	0.52 (0.22–1.36)

### 2. Analysis of the echocardiographic subgroup

Baseline echocardiography was performed in 157 patients. Demographic characteristics, bioimpedance-derived and echocardiographic parameters of this cohort are described in Table 5. Most importantly, there were no significant differences between the entire (N = 221) group and the echocardiographic subgroup in regard to any of the parameters evaluated in the study (Table 5).

**Table 5:** Demographic characteristics, bioimpedance and echocardiography assessment of the echocardiographic subgroup and of the overhydrated and normohydrated patients (using the RFO = 17.4% cut-off).

	All patients (N = 157)	P*	RFO < 17.4% (N = 135)	RFO > 17.4% (N = 22)	P^#^
Age, years	53.1±12.9	0.64	53.6±12.7	50.1±14.5	0.25
Male, N (%)	81 (51.6)	0.86	64 (47.4)	17 (77.3)	**0.01**
Dialysis vintage, months	85.2 (55.9–134.9)	0.39	82.0 (51.8–131.5)	117.6 (73.4–149.3)	**0.02**
Diabetes, N (%)	13 (8.3)	0.49	13 (9.6)	0 (0.0)	0.22
BMI, kg/m^2^	24.9±4.3	0.52	25.2±4.4	23.1±2.4	**0.002**
Hypertensive, N (%)	108 (68.8)	0.52	92 (68.1)	16 (72.7)	0.67
SBP, mmHg	142.9±15.4	0.87	142.9±15.6	143.6±14.2	0.89
DBP, mmHg	81.4±9.9	0.45	81.4±9.9	81.1±9.3	0.76
CV comorbidities, N (%)	80 (51.0)	0.96	69 (51.1)	11 (50.0)	0.92
CAD, N (%)	37 (23.6)	0.83	34 (25.2)	3 (13.6)	0.24
PVD, N (%)	19 (12.1)	0.87	17 (12.6)	2 (9.1)	0.64
Heart failure, N (%)	58 (36.9)	0.33	50 (37.0)	8 (36.4)	0.95
Stroke, N (%)	7 (4.5)	0.54	6 (4.4)	1 (4.5)	0.98
AFO, L	1.6±1.3	0.81	1.3±1.1	3.6±0.8	**<0.001**
RFO, %	9.5±7.4	0.80	7.7±6.4	20.1±3.1	**<0.001**
TBW, L	34.1±5.9	0.86	33.8±5.9	35.6±5.4	0.16
ECW, L	16.3±2.9	0.75	16.1±2.9	17.8±2.4	**0.01**
ICW, L	17.8±3.3	0.56	17.8±3.3	17.9±3.2	0.84
LTI, %	12.9±2.5	0.27	12.9±2.4	12.7±2.7	0.70
FTI, %	10.6 (7.4–14.9)	0.28	11.0 (7.6–15.4)	8.9 (6.7–10.9)	**0.02**
LVMI (g/m^2.7^)	147.1 (120.5–177.7)	-	147.1 (120.9–178.1)	151.8 (119.7–184.2)	0.79
Interventricular septum, mm	11.5±1.7	-	11.6±1.7	11.3±1.8	0.43
LVPWT, mm	10.7±1.8	-	10.8±1.9	10.6±1.6	0.88
End-diastolic left ventricular diameter, mm	52.0±6.2	-	51.7±5.9	54.1±6.7	0.09
End-systolic left ventricular diameter, mm	32.8±5.3	-	32.6±4.9	34.3±7.1	0.33
LVEF, %	60.3 (57.1–64.5)	-	60.4 (57.3–64.5)	60.0 (56.8–64.5)	0.68
Deaths, N (%)	39 (24.8)	0.28	29 (21.5)	10 (45.5)	**0.02**

Data are expressed as mean ± SD, median with IR, or total number with percentages, as appropriate. Bold values are statistically significant. AFO–absolute fluid overload; BMI–body mass index; CAD–coronary artery disease; CV–cardiovascular; DBP–diastolic blood pressure; ECW–extracellular water; FTI–fat tissue index; ICW–intracellular water; LTI–lean tissue index; LVEF–left ventricular ejection fraction; LVMI–left ventricular mass index; LVPWT–left ventricular posterior wall thickness; PVD–peripheral vascular disease; RFO–relative fluid overload; SBP–systolic blood pressure; TBW–total body water.

*—comparison between the entire and the echocardiographic cohort

^#^—comparison between groups.

During the follow-up period (median 68.4 months, IR = 47.8–70.3 months) 39 deaths and 60 CVE were recorded. In the univariate Cox survival analysis, a RFO>15% was associated with an increased risk for all-cause mortality and CVE (HR = 1.91, 95%CI = 1.00–3.64 and HR = 1.83, 95%CI = 1.07–3.12). Importantly, after adjustment for age, gender, dialysis vintage, diabetes, cardiovascular comorbidities, hypertension, and echocardiographic parameters (LVMI or LVEF as continuous variables) these associations were lost. However, performing the same analysis with the 17.4% RFO cut-off, showed that patients in the overhydrated group (RFO>17.4%) had an increased risk for both outcomes, in the univariate and also in the fully adjusted Cox models (Table 6). Using LVEF as a categorical variable didn’t influence the final results of the Cox analysis (S3 Table).

**Table 6:** Survival analysis using the new cut-off of RFO (17.4%) in the echocardiographic subgroup.

	All-cause mortality		Cardiovascular events	
	HR^a^	95% CI	HR^a^	95% CI
Unadjusted	2.27	1.11–4.66	3.26	1.85–5.75
Model 1	2.19	1.02–4.69	3.99	2.13–7.46
Model 2	2.29	1.08–4.89	4.32	2.32–8.05

a. The group of patients with a RFO≤17.4% was used as reference.

**Model 1**: adjusted for age, gender, dialysis vintage, diabetes, cardiovascular comorbidities, hypertension, and left ventricular mass index

**Model 2**: adjusted for age, gender, dialysis vintage, diabetes, cardiovascular comorbidities, hypertension, and left ventricular ejection fraction.

## Discussion

The present study shows that hydration status evaluated by bioimpedance spectroscopy is associated, independently of clinical and echocardiographic parameters, with all-cause mortality and CVE in a HD population. In addition, we also show that overhydration is associated with an increased incidence rate for all-cause hospitalizations. More importantly, we identified a better cut-off to optimally define the increased risk for these outcomes associated with overhydration. To our knowledge this is the first study that evaluates mortality, CVE and hospitalization risk associated with overhydration, taking into consideration different cardiac parameters (comorbidities plus baseline echocardiography).

Overhydration has been identified as an independent predictor for death in dialysis populations. Since the clinical evaluation of dry weight in HD patients is confounded by different patients-specific issues (arterial stiffness, cardiac abnormalities, malnutrition, comorbidities), the bioimpedance analysis (as a more objective and reproducible bed-side diagnostic tool), could help in this essential assessment [15]. Initial prospective observational studies have shown the usefulness of bioimpedance in evaluating and optimizing the correct hydration status of dialysis patients. Moving patients into the normohydration target range leads to a better control of hypertension [7, 8], less intradialytic adverse events and improved cardiac function[7, 16].

Currently, bioimpedance is routinely used in a growing number of dialysis centers but the operational threshold for (a RFO > 15%) relative overhydration is based on the single prospective observational study performed by Wissemann et al. in a 269 prevalent HD patient cohort [12]. Although our cohort included younger patients (mean age 53.8 vs. 65 years) with a longer dialysis vintage and a lower prevalence of diabetes (10.4% vs. 28%), the bioimpedance assessment of fluid compartments was similar and yielded a similar prognostic significance. Using a different statistical approach, we defined a new cut-off for RFO – 17.4% and showed that this value performs better than the previous one in survival analyses. Most importantly, this new threshold remains independently associated with mortality, even after adjustment for different baseline echocardiographic parameters–essential confounders for mortality risk predictions, as shown repeatedly in previous studies [17–21]. Thus, besides confirming data from a single previous cohort, we now robustly bring proof that bioimpedance parameters are significant predictors for outcomes, even when adjustments are made for cardiovascular comorbidities or for well-recognized predictors such as LVMI or cardiac function.

Despite the increasing number of reports and use of bioimpedance for routine dialysis management, in fact few randomized controlled trials compared this bedside method with usual clinical methods of fluid assessment. The first trial was published by Hur et al. and showed that over 12 months, the exclusive use of bioimpedance for determining the dry weight leads to a significant decrease in BP values, arterial stiffness and ultimately LVMI [9]. The second randomized trial confirmed that strict bioimpedance guided fluid management leads to a better BP control, a decrease in arterial stiffness and most importantly, a better survival [10]. Although these randomized trials acknowledge without a doubt the usefulness of the bioimpedance analysis for the dry weight assessment in HD patients, they do not properly define the intimate relationship between overhydration, cardiac function and survival. There is a direct link between hydration status and cardiac function, but whether the increased mortality associated with overhydration is mediated through changes in cardiac performance is currently unknown. Our study, although observational, is the first one to tackle on this endeavor, suggesting that overhydration is linked to mortality independently of cardiac function.

Our study has several limitations. First, echocardiography was not performed in all patients that had the bioimpedance assessment, but such a scenario is usually encountered in similar studies due to inherent patient’ and operator limitations. Also, transthoracic echocardiography is less performant for evaluating cardiac function and structure when compared to other imagistic methods (eg. MRI). Second, we did not assessed residual renal function as this parameter could have influenced body fluid composition, but the high dialysis vintage observed in our study implies that most of the patients were anuric. Third, the number of patients and outcome events were relatively low, but we used different statistical approaches to robustly overcome these shortcomings. Fourth, our patients come from a country with one of the best survival rates in the world [22] and, as such, our results may not be inferred to other populations. Fifth, although LVMI is associated with diastolic dysfunction, as diastolic function is always altered in the presence of left ventricular geometrical remodeling, and also that both LVMI and LVEF are independent and important prognostic factors in HD populations [23, 24], our results cannot be generalized to HD patients with only diastolic dysfunction.

In conclusion, we show that the hydration status is associated with the mortality, CVE and hospitalization risk in a HD population, independently of cardiac morphology and function. We also describe and propose a new cut-off for RFO, in order to better define this relationship between overhydration and worse outcomes. Further studies are needed to properly validate this new cut-off in other HD populations.

## Supporting Information

S1 Table Demographic characteristics and bioimpedance assessment of the overhydrated and normohydrated patients (using the RFO = 17.4% cut-off) from the entire study population (DOCX)

S2 Table Causes of death for the study population. (DOCX)

S3 Table Survival analysis using the left ventricular ejection fraction as a categorical variable in the echocardiographic subgroup. (DOCX)
