# Supplementary material for: Overhydration, Cardiac Function and Survival in Hemodialysis Patients
Source: PLoS One. 2015 Aug 14;10(8):e0135691. doi: 10.1371/journal.pone.0135691 (PMC4537261; doi:10.1371/journal.pone.0135691)
Supplement: S1 Table — (DOCX) [file pone.0135691.s001.docx]

**Supplementary Table 1.** Demographic characteristics and bioimpedance assessment of the overhydrated and normohydrated patients (using the RFO=17.4% cut-off) from the entire study population

|  | RFO ≤ 17.4%  N=181 | RFO > 17.4%  N=40 | P |
| --- | --- | --- | --- |
| Age, years | 53.9±13.6 | 52.9±15.0 | 0.65 |
| Male, N (%) | 88 (48.6) | 28 (70.0) | **0.01** |
| Dialysis vintage, months | 77.2  (46.3-123.6) | 108.7  (72.4-148.8) | **0.002** |
| Diabetes, N (%) | 17 (9.4) | 6 (15.0) | 0.29 |
| BMI, kg/m^2^ | 25.9±5.2 | 23.9±3.9 | **0.01** |
| Hypertensive, N (%) | 116 (64.1) | 29 (72.5) | 0.31 |
| SBP, mmHg | 143.3±15.7 | 142.1±16.0 | 0.68 |
| DBP, mmHg | 80.9±10.3 | 78.5±10.1 | 0.18 |
| CV comorbidities, N (%) | 91 (50.3) | 21 (52.5) | 0.79 |
| CAD, N (%) | 45 (24.9) | 5 (12.5) | 0.09 |
| PVD, N (%) | 24 (13.3) | 4 (10.0) | 0.62 |
| Heart failure, N (%) | 57 (31.5) | 14 (35.0) | 0.67 |
| Stroke, N (%) | 9 (5.0) | 4 (10.0) | 0.26 |
| AFO, L | 1.2±1.1 | 3.7±0.8 | **<0.001** |
| RFO, L | 7.12±6.4 | 21.2±3.5 | **<0.001** |
| TBW, L | 33.9±6.1 | 34.6±5.9 | 0.66 |
| ECW, L | 16.2±2.9 | 17.5±2.7 | **0.01** |
| ICW, L | 17.7±3.4 | 16.9±3.4 | 0.13 |
| LTI, Kg/m^2^ | 12.7±2.5 | 11.9±2.6 | 0.09 |
| FTI, Kg/m^2^ | 11.9  (8.4-15.6) | 9.4  (6.7-13.2) | **0.02** |
| Deaths, N (%) | 44 (24.3) | 22 (55.0) | **<0.001** |
| CVE, N (%) | 51 (28.2) | 27 (67.5) | **<0.001** |

Data are expressed as mean ± SD, median with IR, or total number with percentages, as appropriate. Bold values are statistically significant. AFO – absolute fluid overload; BMI – body mass index; CAD – coronary artery disease; CV – cardiovascular; CVE – cardiovascular events; DBP – diastolic blood pressure; ECW – extracellular water; FTI – fat tissue index; ICW – intracellular water; LTI – lean tissue index; PVD – peripheral vascular disease; RFO – relative fluid overload; SBP – systolic blood pressure; TBW – total body water.

^#^ - comparison between groups
