# Supplementary material for: Overhydration, Cardiac Function and Survival in Hemodialysis Patients
Source: PLoS One. 2015 Aug 14;10(8):e0135691. doi: 10.1371/journal.pone.0135691 (PMC4537261; doi:10.1371/journal.pone.0135691)
Supplement: S2 Table — (DOCX) [file pone.0135691.s002.docx]

**Supplementary Table 2.** Causes of death for the study population.

|  | Entire Cohort | Echocardiography subgroup |
| --- | --- | --- |
| Cardiovascular, N (%) | 30 (45.5) | 17 (43.5) |
| Sudden death, N (%) | 20 (30.3) | 12 (30.8) |
| Sepsis, N (%) | 13 (19.7) | 8 (20.5) |
| Neoplasm, N (%) | 1 (1.5) | 1 (2.6) |
| Cirrhosis, N (%) | 2 (3.0) | 1 (2.6) |
| **Total** | 66 | 39 |
