# Supplementary material for: Overhydration, Cardiac Function and Survival in Hemodialysis Patients
Source: PLoS One. 2015 Aug 14;10(8):e0135691. doi: 10.1371/journal.pone.0135691 (PMC4537261; doi:10.1371/journal.pone.0135691)
Supplement: S3 Table — (DOCX) [file pone.0135691.s003.docx]

Supplementary Table 3. Survival analysis using the left ventricular ejection fraction as a categorical variable in the echocardiographic subgroup.

|  | All-cause mortality | | Cardiovascular events | |
| --- | --- | --- | --- | --- |
|  | HR | 95% CI | HR | 95% CI |
| Model 1^a,b^ | 1.82 | 0.92-3.59 | 1.80 | 0.97-3.17 |
| Model 2^a,b^ | 2.37 | 1.12-5.04 | 4.06 | 2.19-7.53 |

a. Adjusted for age, gender, dialysis vintage, diabetes, cardiovascular comorbidities, hypertension, and left ventricular ejection fraction.

b. The group of patients with a RFO≤15 %, and was used as reference.

c. The group of patients with a RFO≤17.4 %, and was used as reference.
